# Veterinary Pathology – A Path Forward with New Directions and Opportunities

**DOI:** 10.3389/fvets.2016.00076

**Published:** 2016-08-31

**Authors:** Tracy Stokol

**Affiliations:** ^1^Department of Population Medicine and Diagnostic Sciences, College of Veterinary Medicine, Cornell University, Ithaca, NY, USA

**Keywords:** diagnostic assays, imaging modalities, wildlife disease, emerging disease, nanomedicine, consensus statements, genome-wide association studies

As veterinary pathologists, we have a broad knowledge of disease processes in an array of species with variable physiology and response to disease. The breadth of areas where veterinary pathologists are active has led us to sub-specialize in particular species, organ systems, infectious agents, or sub-disciplines, such as forensic science. We are a group with tremendously diverse interests and expertise that includes basic and clinically applied research, drug discovery and preclinical toxicologic testing, diagnostic testing and quality assurance, public health and governmental policy, public outreach, and education. Our diversity of interests is both a strength and a challenge. Should we strive to maintain a unified discipline or divest into defined subspecialties? How should we define ourselves as pathologists? What core skill sets and competences should we include for certification and how do we prepare our trainees for the future? Should we have one international certification? Finally, how do we make our trainees aware of the many opportunities available to them?

The American College of Veterinary Pathologists is addressing these challenges in part by revising the structure and content of the specialty board examination. A working group has also been established to develop a “strategic plan for the future.” This group convened at the 2015 annual meeting and, in its initial discussions, recognized that (1) veterinary pathologists must adapt and respond to the changing needs for our stakeholders and (2) a renewed importance should be placed on research in training programs.^1^ Although the working group is likely several years away from delivering final recommendations, it seems likely that emphasis will be placed on research training. There is currently no stipulation for a research component in residency programs. In my view, building the infrastructure to support investigative research as an integral part of our training programs would be a positive step forward. By expanding our knowledge and informing future diagnostic practice, research will help keep our discipline relevant, vital, and current. I believe that training in investigative research will stimulate critical thinking in our trainees and potentially increase their competitiveness and the qualified applicant pool for faculty and leadership positions. It will also help feed the intellectual curiosity that drives us.

Our field is defined by the juxtaposition of descriptive and mechanistic explorations of disease processes. Identifying abnormalities and characterizing known and emerging diseases is a critical facet of what we do. Salient examples include the pathologist’s role in recognizing the emergence of West Nile virus in the United States (1) and elucidating the fungal cause of white nose syndrome in bats (2). This descriptive research is an essential part of diagnostic pathology. Yet, we, as pathologists, also study the step-by-step development of disease from the molecular and genetic level to the animal level. Our profession strives to develop tools to identify disease at its earliest stage, when interventions are more likely to be successful. This effort is crucial to global health, as diseased animals affect their environment causing downstream ecological effects on humans, plants, and other animals. To help us explore disease at all these levels and the complex interrelationships between animal and human health, I believe we should continue to take advantage of new and emerging disciplines, incorporating them into our investigational pursuits, as outlined below. We can also forge new cross-disciplinary partnerships and become leaders and essential contributors in collaborative teams. We should strive to ask novel questions and challenge dogma in animal disease that will help inform human studies and increase our competitiveness with national funding agencies.

## Epigenetics and Genetics

The genomes of several animal species have been sequenced and annotated, including dogs (3), cats,^2^ and horses.^3^ These resources have allowed us to employ genome-wide association mapping to identify genetic abnormalities in inbred animals with simple disease traits that have helped inform similar diseases and normal development in humans (4–6). These breakthroughs have in turn resulted in the development of diagnostic tests for the genetic traits, which has potential for reducing disease prevalence (6–8). Genetic screening has also revealed associations with specific loci for complex diseases (9). Further investigation of these loci may lead us to discover causative genes and develop relevant diagnostic tests. Despite the rapid advances in genomic mapping, many challenges remain. For example, we are just beginning to explore epigenetic patterns in animals (10, 11) but lack important tools, such as promoter arrays. We also need more information on cancer genomes in animals. I expect that these tools will eventually be developed. Our discipline must be ready to incorporate these advances in genomics into our training and diagnostic programs.

## Nanoengineering

The burgeoning field of biomedical engineering has rapidly morphed from basic research into translational medicine, with the application of engineering principles to cancer diagnostics, drug delivery, imaging, and infectious disease detection, to name a few applications (12–15). This field, together with genomic analysis, has ushered in personalized medicine in humans. Application of nanoengineering tools has just begun in veterinary medicine (16–18), but the goal of personalized medicine for pet animals is likely to come sooner rather than later. As pathologists, we hold a unique position where we can be both leaders and an integral member of multidisciplinary teams in the charge toward personalized medicine.

## Diagnostic Imaging

The introduction of powerful advanced techniques, such as imaging flow cytometry (19–21) and histology-directed imaging mass spectrophotometry (22), is yielding new insights into disease pathogenesis. As dedicated instruments become more commonplace and cost-effective, it will only be a matter of time before these technologies enter the diagnostic realm. I, for one, am excited by the opportunity that these modalities represent. Pinpoint accuracy for infectious disease diagnosis, novel insights into biochemical pathways that lead to pathology – the possibilities are endless.

## Consensus Statements and Standardization of Testing

Unlike human medicine, our diagnostic activities are not federally regulated. Veterinary diagnostic laboratories do not have to follow Clinical Laboratory Improvement Amendments regulations.^4^ Nevertheless, many laboratories voluntarily participate in external proficiency testing, with recommendations for this testing being established by the American Society of Veterinary Clinical Pathology (23). Guidelines and consensus statements have been generated for several diagnostic activities, including reference interval establishment (24), prognostic markers in cancer (25), flow cytometric reporting in canine hematopoietic neoplasia (26), immunocytochemical staining (27), and viscoelastic-based hemostasis testing (28), to name a few examples. Similarly, cytologic (29) and histologic (30, 31) grading schemes have been proposed for various tumors. Importantly, these guidelines are the culmination of efforts of multiple investigators and truly represent a global approach (“One Pathology”). Their frequency of citation and routine application during diagnostic testing attests to the usefulness of these guidelines. I believe that there is a clear need for new guidelines for our vast array of assays, such as histopathologic tumor grading, clonality assays and reporting, and flow cytometric methods. Ideally, these guidelines should be grounded in evidence-based medicine and have a built-in plan for re-evaluation. Up-to-date guidelines are crucial for standardization and allow cross-laboratory comparison of results, increasing the usefulness of results and our confidence in the provided data. There is also a need for increased availability of affordable veterinary-specific proficiency testing. This is particularly pertinent for assays that use species-specific reagents, such as flow cytometry and clonality testing. In the absence of enforced regulation, it falls to us, the pathologists, and our Colleges and Societies, to generate more of these consensus statements or recommendations for standardization of testing and reporting.

## Human–Wildlife Boundaries

Human activity continues to encroach upon uninhabited areas perturbing ecosystems and wildlife populations. A consequence of bringing human populations into close contact with previously isolated wild species is the interspecies transmission of infectious disease. Recently, we have seen outbreaks of Ebola and Zika viruses in humans, viruses that have previously been largely restricted to wildlife (32, 33). Conversely, viruses that are typically considered pathogens of domestic animals, such as canine distemper, have spread into naive wildlife, with devastating effects (34). Technologic advances, such as hydrofracking, have allowed for the increased utilization of previously inaccessible natural resources. Yet, these come at a health cost to animals and humans (35). Wildlife species are sentinels of environmental changes, the proverbial “canary in the coal mine.” The emergence and spread of fungal diseases, such as *Pseudogymnoascus destructans* (2) and chytridiomycosis (*Batrachochytrium dendrobatidis*) (36), emphasizes our need to be continually and actively vigilant at screening wildlife for environmental changes that may affect human and animal health. Veterinary pathologists will continue to play a vital and prominent role in identification and investigation of these diseases.

The scope of this section of Frontiers in Veterinary Science is pathology – the study of disease – at a genetic (or epigenetic), molecular, cellular, tissue, organism, and herd level in animals. The scope encompasses the study of factors that contribute to disease, such as the aforementioned environmental changes. Our focus is animal health. Through improving animal health, we improve the health and well-being of humans, whose lives are intricately tied to and reliant on the animals that serve as food, companions, and workers. We accept various manuscript types^5^ and strongly encourage submission of prospective descriptive or hypothesis-driven studies in topics related to pathology. To complement our current research topics, future planned research topics are hematopoietic neoplasia, hemostasis, and wildlife as sentinels of disease.

So why publish in Frontiers in Veterinary Science?

- Open-access: this makes our research available to everyone.
- Rapid publication: with high expectations for productivity for early career pathologists, there is an urgent need for rapid publication. Fast turnaround times are only available through non-traditional publication avenues, such as open-access online journals. The average time from submission to online publication is around 2–3 months for Frontiers.
- Minimal stylistic editing: write your manuscript in active voice and your own style. Tell us your story.
- Copyright: authors retain copyright through a Creative Commons License. If you are a member of Linked-In, Research Gate, Loop, or other professional networks, this allows you to post your articles or use your data without obtaining permission from a publisher.
- A collaborative interactive open review process: this can eliminate the frustrations, misconceptions, and seemingly endless revisions that inevitably accompany one-dimensional reviews. With Frontiers, authors discuss their rationale, clarify points, and address reviewers’ concerns through an online interactive forum with reviewers and the associate editor. Reviewers work with authors to help improve manuscript quality. Should your manuscript be accepted, the names of reviewers and associate editor are published. This encourages constructive, informative critical review.
- Assessment of impact: Frontiers has a tiered impact system, which takes advantage of the best of social networking and media. Impact is not only based on citations but also number of downloads, page views, etc. A question that remains to be answered is: Does this method works better than that already in use?

Our goals with Veterinary Experimental and Diagnostic Pathology are to publish high-quality research that showcases the breadth and strength of veterinary pathology and to advance our discipline. In doing so, we maintain our relevance and inspire our successors. Can we accomplish our goal? Only if you, the investigators, readers, and reviewers support this vision, ask the questions, and conduct the studies, then publish your results after collaborative, respectful, and thoughtful review. Why don’t you join us? I invite you to submit.

## Author Contributions

The author confirms being the sole contributor of this work and approved it for publication.

## Conflict of Interest Statement

The author declares that the research was conducted in the absence of any commercial or financial relationships that could be construed as a potential conflict of interest.

## Footnotes

^1^http://www.acvp.org/PDF/2016_ACVP_June_Newsletter_WEB.pdf

^2^http://www.ncbi.nlm.nih.gov/genome?term=felis%20catus

^3^http://www.uky.edu/Ag/Horsemap/

^4^https://wwwn.cdc.gov/clia/

^5^http://journal.frontiersin.org/journal/veterinary-science#article-types
